# Chemical Composition and Synergistic Antimicrobial Effects of Essential Oils From Four Commonly Used *Satureja* Species in Combination With Two Conventional Antibiotics

**DOI:** 10.1002/cbdv.202402093

**Published:** 2025-03-15

**Authors:** Imane Abbad, Bouchra Soulaimani, Marcello Iriti, Mustapha Barakate

**Affiliations:** ^1^ Laboratory of Water Sciences, Microbial Biotechnologies, and Natural Resources Sustainability (AQUABIOTECH), Unit of Microbial Biotechnologies, Agrosciences, and Environment (BIOMAGE)‐CNRST Labeled Research Unit No. 4, Faculty of Sciences‐Semlalia University Cadi Ayyad Marrakech Morocco; ^2^ Department of Biomedical, Surgical and Dental Sciences Università degli Studi di Milano Milan Italy; ^3^ National Interuniversity Consortium of Materials Science and Technology Florence Italy

**Keywords:** antibiotics, antimicrobial activity, essential oils, *Satureja* spp, synergism

## Abstract

The chemical composition and the antimicrobial potency of four *Satureja* essential oils (EOs), and their synergism with two antimicrobials have been investigated. Gas chromatography (GC) and GC/mass spectrometry analysis showed that *S. alpina* EO was predominated by pulegone (88.8%), while pulegone (38.6%) and menthone (30%) were the major constituents of *S. calamintha* EO. *S. montana* and *S. hortensis* EOs were dominated by carvacrol (50.8%–32.8%), γ‐terpinene (18.5%–40.1%), and p‐cymene (8.2%–7.3%), respectively. The antimicrobial activity showed that *S. montana* and *S. hortensis* EOs exhibited potent activity (minimal inhibitory concentration and minimum microbiocidal [bactericidal and candidacidal] concentrations = 0.07–4.46 mg/mL for bacteria, and 0.27–1.11 mg/mL for Candida). All EOs showed high synergism with gentamicin against bacteria (gains ranged between 4‐ and 512‐fold). Interestingly, this synergism was pronounced against the Gram‐negative bacteria *Escherichia coli* and *Pseudomonas aeruginosa*. Regarding the association with amphotericin B, synergistic and additive effects were recorded depending on the strain tested.

## Graphical abstract

The chemical composition and the in vitro synergistic antimicrobial effects of four Satureja essential oils were investigated. Essential oils from Satureja montana and S. hortensis displayed strong antimicrobial effects, whereas S. calamintha and S. alpina exhibited relatively weaker antimicrobial activity. Interestingly, all tested Satureja essential oils demonstrated significant synergy when combined with the antibiotic gentamicin. More details can be found in article number e202402093 by Imane Abbad and co‐workers.

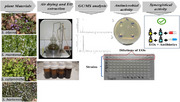

## 1 Introduction

Antimicrobial resistance (AMR) has been described as a major risk in healthcare systems and is emerging as a problem in community‐acquired infections [1, 2]. AMR has been ranked by the World Health Organization (WHO) among the top 10 global public health hazards facing mankind in which conventional antimicrobials turn out to be ineffective in the treatment of infections for which they were specially conceived [3]. Many previous reports highlighted the negative economic and clinical impacts of AMR. In fact, it has been estimated that annually 700 000 persons die worldwide due to infections caused by multi‐drug resistant bacteria, and there is no doubt that this number will increase in the coming years [4, 5]. In 2019, the United Nations Interagency Coordination Group on AMR alerted that, in the absence of efficient strategies, antibiotic‐resistant diseases could cause annually 10 million deaths by 2050, with a projected cost to be around 100 trillion dollars, and could force up to 24 million people into extreme poverty [6]. Faced with this alarming situation, the development of novel antimicrobial agents to control AMR seemed to be an urgent global need. Recently, in the absence of new effective drugs and to counteract this AMR, many studies and reviews have highlighted the potential application of some plant‐based extracts (e.g., essential oils [EOs]) as promising alternative biobased antimicrobials [7, 8, 9]. In fact, several reports pointed out the interesting antimicrobial properties of some plant EOs against a broad spectrum of pathogenic microorganisms, mainly due to their lipophilicity and their phytochemical diversity [10]. These characteristics led to EO components easily passing through the phospholipid bilayer of the bacterial cell membrane and interacting with different cellular constituents, affecting several metabolic and physiological processes that are crucial for cell survival [11, 12]. In addition to their simultaneously multiple target sites in microbial cells, EOs as a source of bioactive ingredients, have the cumulative advantage of being biodegradable, cost‐effective, eco‐friendly, and with no adverse side effects [12, 13]. Regarding these properties and in order to tackle the development of resistance, many studies have investigated the possible synergism between effective EOs and antibiotics [14, 15, 16]. This approach has proven effective in overcoming bacterial resistance mechanisms, thereby increasing the susceptibility of pathogens to antibiotics. In fact, many studies have shown that certain components of EOs can inhibit bacterial efflux pumps, which are typically responsible for expelling antibiotics from cells, thereby potentially enhancing the drug's effectiveness [7, 9, 10]. Additionally, because EOs can target multiple cellular sites, they can produce a more pronounced overall impact [17]. Thus, this combination antibiotic therapy has been suggested as a promising and powerful strategy to restore the efficacy of some conventional antimicrobials to resistant pathogenic microorganisms [16].

The genus *Satureja* (Lamiaceae) includes various aromatic and medicinal species that are largely used as valuable ingredients for food, cosmetics, and pharmaceutical industries [18]. Of these *Satureja* species, *S. hortensis*, *S. montana*, *S. alpina*, and *S. calamintha* are of great economic importance due to their richness in EOs, which are traditionally exploited in Mediterranean regions to treat various infectious illnesses. Several previous works have highlighted the interesting antimicrobial properties of some *Satureja* EOs, including the winter savory EO (*S. montana*) [19, 20]. However, little information is available on the antimicrobial potency of other commonly used *Satureja* species and their potential synergism with antibiotics. Thus, the present works aimed to compare the antimicrobial activity of EOs obtained from these four *Satureja* species and to investigate their potential synergistic combinations with two well‐known antibiotics (gentamicin and amphotericin B) against a panel of pathogenic bacteria and candida.

## 2 Results and Discussion

### 2.1 EO Yields and Chemical Composition

**FIGURE 1:**
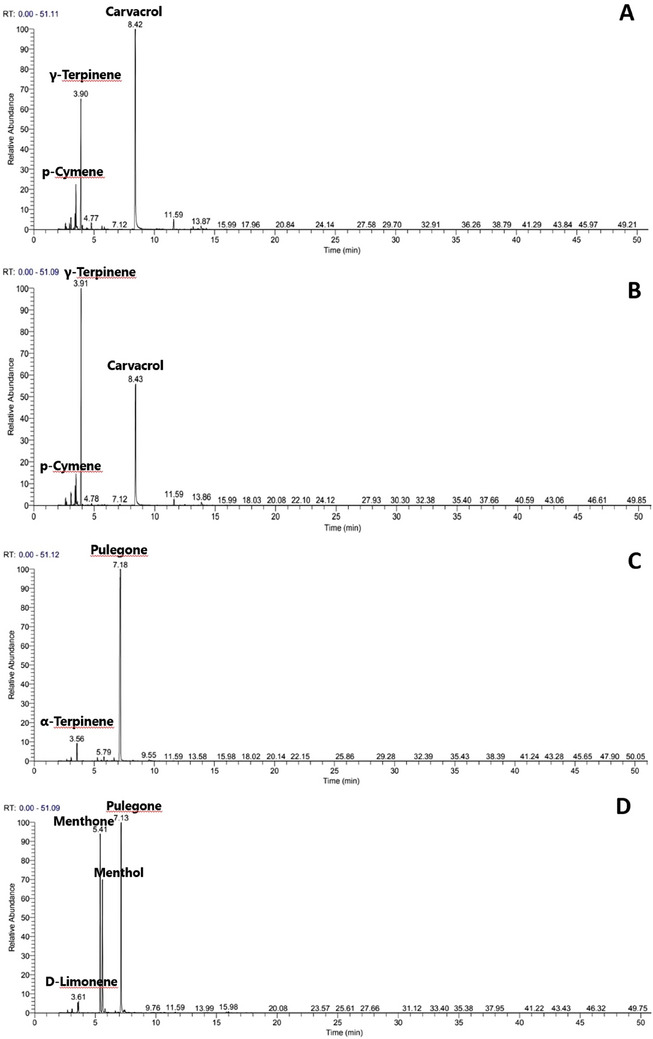
Chromatographic profiles of four *Satureja* species: (A) *Satureja montana*, (B) *Satureja hortensis*, (C) *Satureja alpina*, and (D) *Satureja calamintha*.

The EOs obtained from aerial parts of studied *Satureja* species through hydro‐distillation were observed to be of pale‐yellow color, with variable yields ranging from 0.42 ± 0.03% to 2.14 ± 0.12 % (w/w) based on dry weight (Table 1). The highest oil yield was observed in *S. montana*, while *S. alpina* yielded the lower value (Table 1). The results of the chemical analysis of the volatile constituents of *Satureja* EOs with the percentage content of each compound and structural subclass are presented in Figure 1 and Table 2. Twenty‐six compounds were identified, accounting for 96.2%–98.76% of the total EO constituents. The studied *Satureja* EOs were quantitatively dominated by the oxygenated monoterpenes class (56.6%–93.5%), except *S. hortensis* EO which was dominated by the group of monoterpene hydrocarbons (57.3%), then oxygenated monoterpenes (34.6%). GC and GC/MS analysis revealed a high content of the monoterpene pulegone (88.2%) in *S. alpina* EO, while pulegone (38.6%), menthone (30%) and menthol (21.2%) were identified as major constituents of *S. calamintha* EO, which is consistent with previous studies conducted on specimens originating from the Moroccan origin [21, 22]. Nonetheless, other EO profiles characterized by high levels of 1,8‐cineole, β‐phellandrene, and pinocamphone have been documented for this species obtained from northern Morocco [23]. Concerning *S. alpina* EO, a high content of monoterpene pulegone has been identified, aligning with chemical profiles observed in EOs from other Moroccan samples [24, 25]. The EOs of *S. montana* and *S. hortensis* were dominated essentially by the phenolic monoterpene carvacrol (50.8% and 32.8%) followed by γ‐terpinene (18.5% and 40.1%) and p‐cymene (8.2% and 7.3%), respectively. These findings align closely with previously reported profiles for these aromatic species [19, 20, 26, 27]. However, in some studies [28, 29, 30], thymol has been consistently identified either along with these three components or as the primary constituent in EOs obtained from different other samples. It's noteworthy that both thymol and carvacrol have been established as distinctive components within the species' chemotypes, indicating substantial chemical polymorphism within these species [31, 32, 33].

**TABLE 1:** Locality, harvesting location and period, voucher specimens, and EO yield of the four *Satureja* species studied.

Species	Local name	Harvesting place	Harvesting time	Voucher specimens	Latitude/Longitude	Oil yield^a^ (mg / 100 g)
*S. calamintha*	Minta	Oukaimeden	July 2022	SATCA019	31°11′N/07°53′W	1.53 ± 0.13
*S. alpina*	Fliou dial lbar	Oukaimeden	July 2022	SATAL057	31°13′N/07°53′W	0.42 ± 0.03
*S. montana*	Zaater erroumi	Oulad Dlim	September 2022	SATMO017	32°01’N/08°13’W	2.14 ± 0.12
*S. hortensis*	Zaater erroumi	Oulad Dlim	September 2022	SATHO012	32°01’N/08°14’W	1.25 ± 0.04

^a^ yield of EOs determined based on their weight / 100 g of dried plant used for distillation.

**TABLE 2:** Chemical compositions of studied *Satureja* EOs.

RT^a^	RI^b^	RI^c^	Compounds	*S. montana*	*S. hortensis*	*S. calamintha*	*S. alpina*
2.67	968	924	β‐Thujene	1.3	1.7	—	0.4
2.76	973	932	α‐Pinene	—^d^	1.1	0.5	0.1
3.01	980	969	Sabinene	1.2	—	0.1	0.2
3.15	981	990	α‐Myrcene	2.2	2.6	0.1	0.7
3.51	1010	1014	α‐Terpinene	3.1	4.5	0.7	—
3.60	1016	1020	p‐Cymene	8.2	7.3	—	—
3.61	1031	1024	D‐Limonene	—	—	1.6	3.9
3.72	1035	1026	1,8‐Cineole	—	1.2	1.6	—
4.02	1047	1054	γ‐Terpinene	**18.5**	**40.1**	—	0.1
4.15	1056	1098	trans‐Sabinene hydrate	1.1	—	—	—
4.91	1101	1112	trans‐Thujone	1.6	0.7	—	—
5.41	1136	1148	Menthone	—	—	**30.0**	0.8
5.59	1155	1167	Menthol	—	—	**21.2**	0.3
5.80	1160	—	trans‐Menthone	—	—	1.0	1.6
5.84	1162	1165	Borneol	1.1	—	—	—
6.03	1164	1174	Terpinen‐4‐ol	1.0	—	—	0.3
6.08	1182	1179	Isomenthol	—	—	0.1	0.9
7.13	1237	1233	Pulegone	—	—	**38.6**	**88.2**
7.43	1240	1316	Citral	1.0	—	0.4	0.1
8.4	1298	1298	Carvacrol	**50.8**	**32.8**	—	—
8.70	1335	1340	Piperitone	—	—	0.6	0.4
11.96	1419	1417	Caryophyllene	2.7	2.1	2.0	0.6
13.59	1477	1478	γ‐Muurolene	1.6	—	—	—
14.01	1494	1484	Germacrene D	0.9	—	—	—
14.22	1531	1506	α‐Bisabolene	2.2	2.2	—	—
15.98	1601	1582	Caryophyllene oxide	—	—	0.3	0.1
Oxygen‐containing monoterpenes				56.6	34.6	93.5	92.6
Monoterpene hydrocarbons				34.5	57.3	3.0	5.4
Oxygen‐containing sesquiterpenes				—	—	0.3	0.1
Sesquiterpene hydrocarbons				7.4	4.3	2.0	0.6
Total				98.5	96.2	98.8	98.7

^a^ RT: retention time.

^b^ RI: Retention index relative to n‐alkanes (C7‐C30) on the TG‐5MS capillary column.

^c^ RI: Retention indices from literature (Adams, 2007).

^d^ not detected.

The values in bold are the dominant compounds.

### 2.2 Antimicrobial Activity of *Satureja* EOs

**FIGURE 2:**
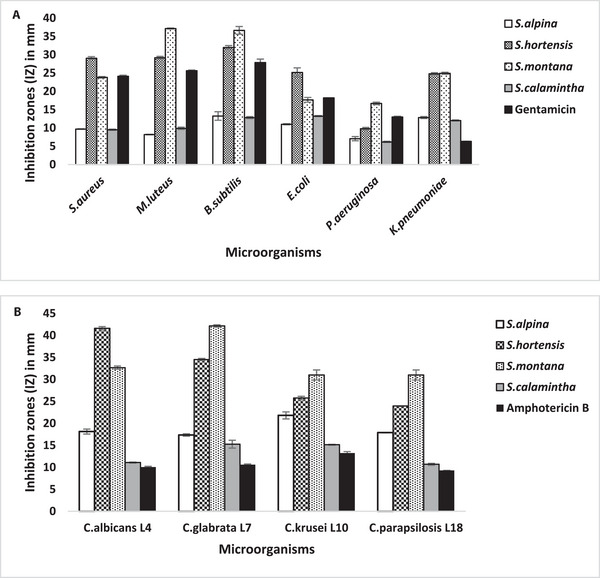
Inhibition‐zone diameters (IZ mm) determined with the disc‐diffusion method of the four *Satureja* essential oils (EOs) and antibiotics against bacterial (A) and candida (B) strains tested (Inhibition zone diameters include a disc diameter of 6 mm with a concentration of 10 µL of oil per disc, 15 µg of gentamicin per disc, and 5 µg of amphotericin B per disc).

**FIGURE 3:**
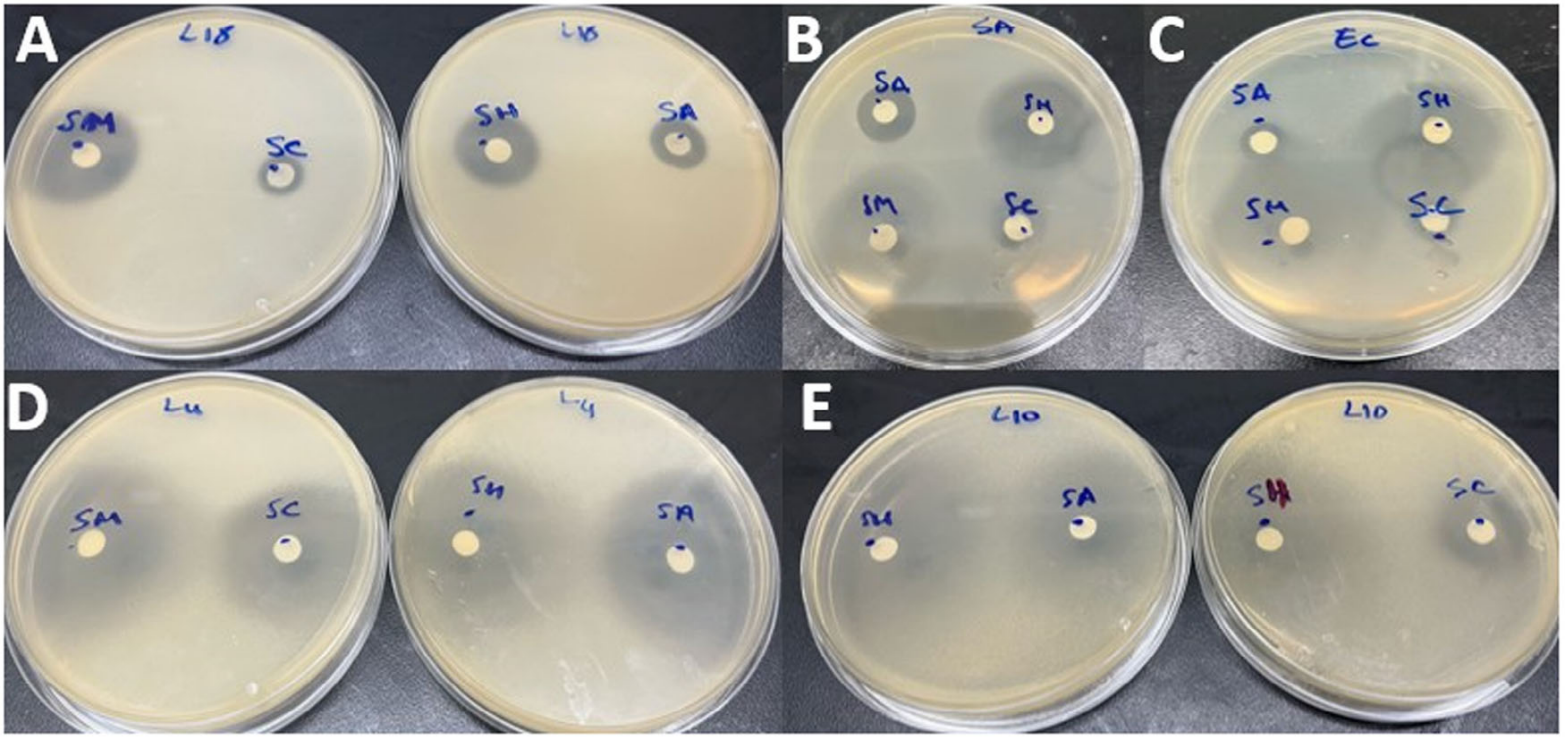
Inhibition zone diameters of candida and bacterial strains for four *Satureja* species: (A) *Candida parapsilosis*, (B) *Staphylococcus aureus*, (C) *Escherichia coli*, (D) *Candida albicans*, and (E) *Candida krusei*. SA: *S. alpina*, SC: *S. calamintha*, SM: *S. montana*, and SH: *S. hortensis*.

The zones of microbial growth inhibition were measured in order to assess the initial antimicrobial screening of studied *Satureja* EOs. The findings showed variable inhibition zone (IZ) diameter values, ranging from 6.18 ± 0.14 to 37.14 ± 0.13 mm for the tested bacteria and from 10.69 ± 0.21 to 42.16 ± 0.24 mm for the tested Candida strains (Figure 2). The EOs extracted from *S. montana* and *S. hortensis* showed the most significant inhibitory effect against all the tested strains, with IZ values reaching up to 42.16 ± 0.24 mm (Figure 3). These values were generally greater than those produced by the reference antimicrobials gentamicin and amphotericin B. In terms of the minimal inhibitory concentration (MIC) and minimum microbiocidal (bactericidal and candidacidal) concentrations (MMC) results, the findings align closely with the observed IZs, thereby reaffirming the previously reported antimicrobial effectiveness of these two aromatic plants [19, 20, 23, 25, 27]. Comparatively, the EO extracted from *S. montana* exhibited the highest potency, with MIC = MMC values within the range of 0.07–1.14 mg/mL, followed by the *S. hortensis* EO values, demonstrating notable efficacy with MICs and MMCs ranging from 0.28 to 4.46 mg/mL (Table 3). The EO from *S. montana* distinguished itself among the four oils investigated due to its interesting bactericidal activity at a low concentration (MIC = MMC = 0.57 mg/mL) against the Gram‐negative *Pseudomonas aeruginosa*. This strain demonstrated greater resistance to other *Satureja* EOs, especially *S. calamintha* and *S. alpina*. However, this activity was lower than that of the antibiotic gentamicin. The interesting activity observed in *S. montana* and *S. hortensis* EOs can primarily be attributed to the abundance of highly bioactive antimicrobial compounds, particularly carvacrol, γ‐terpinene, and p‐cymene, prevalent in these two EOs [34]. Indeed, the antimicrobial effectiveness of these monoterpenoids has been demonstrated in many previous reports, with the phenolic compound carvacrol notably displaying higher activity in comparison to other monoterpenoids [31, 32, 33]. This can explain the comparatively higher activity observed in the EO from *S. montana*, given its elevated proportion of carvacrol compared to that found in *S. hortensis*. Indeed, the hydroxyl group, a distinctive element in the phenolic structure of carvacrol, plays a crucial role in enhancing its effectiveness. The antimicrobial action of carvacrol has been extensively documented, including its capability to disrupt microbial cell membranes. Specifically, carvacrol interferes with the lipid bilayer, inducing structural alterations that heighten permeability and ultimately result in cell lysis. Furthermore, it has been noted to impede ATPase activity, essential for cellular energy production, thereby augmenting the inhibition of microbial growth [32, 35, 36]. Regarding the antimicrobial activity of *S. calamintha* and *S. alpina* EOs, less pronounced effects have been observed, with IZ diameters ranging from 6.18 ± 0.14 to 21.81 ± 0.80 mm indicating less to moderate activity. Concerning the MIC values, the *S. calamintha* and *S. alpina* EOs displayed relatively promising effects against Candida strains (MIC range: 2.23–4.47 mg/mL), while their activity against tested bacteria appeared comparatively weaker. This result is consistent with earlier reported results [37, 38, 39]. The reduced antimicrobial activity observed in these EOs could be linked to their abundance in pulegone, a monoterpene ketone known for its lower effectiveness when compared to phenolic monoterpenes [40]. Overall, the MMC values for all the studied EOs either equaled or showed minimal variation compared to their MICs, confirming their substantial microbicidal effectiveness.

**TABLE 3:** Minimal inhibition concentration (MIC) and minimal microbicidal concentration (MMC) of tested EOs and antibiotics against six panel of bacterial and four candida strains.

	Essential oils								Antibiotics	
Microorganismes	***S. alpina***		***S. hortensis***		***S. montana***		***S. calamintha***		Gentamicin	Amphotericin B
	MIC	MMC	MIC	MMC	MIC	MMC	MIC	MMC	MIC	MIC
*S. aureus*	36.68	36.68	1.12	2.23	0.29	0.29	35.80	>35.8	0.312	—
*M. luteus*	18.34	18.34	0.28	0.28	0.07	0.07	17.90	17.90	0.625	—
*B. subtilis*	18.34	36.68	0.28	0.28	0.07	0.07	17.90	17.90	0.312	—
*E. coli*	18.34	18.34	1.12	1.12	1.14	1.14	17.90	17.90	5.00	—
*P. aeruginosa*	>36.68	>36.68	4.46	4.46	0.57	0.57	>35.8	>35.8	5.00	—
*K. pneumoniae*	18.34	18.34	0.56	0.56	0.57	0.57	17.90	17.90	160	—
*C. albicans L4*	2.29	9.17	0.28	0.28	0.57	0.57	2.23	2.23	—^a^	3.125
*C. glabrata L7*	2.29	4.58	0.55	0.55	0.57	0.57	2.23	4.47	—	1.56
*C. krusei L10*	2.29	9.17	1.11	1.11	0.28	0.28	4.47	8.95	—	0.78
*C. parapsilosis L18*	2.29	2.29	1.11	1.11	0.57	0.57	4.47	4.47	—	1.56

MIC and MMC are in mg/mL for EOs and in µg/mL for antibiotics; ^a^Not tested.

### 2.3 Synergistic Effect of *Satureja* EOs With Conventional Antimicrobials

The results detailing the synergistic interactions, including MIC gain and fractional inhibitory concentration index (FICI) values of antibiotics, observed between the studied *Satureja* EOs and conventional antimicrobials amphotericin B and gentamicin are given in Tables 4 and 5, respectively. The combinations between *Satureja* EOs and the antifungal amphotericin B exhibited different types of interactions depending on the tested Candida strains. Of the combinations tested, those prepared with *S. alpina* and *S. montana* EOs demonstrated synergistic effects against *Candida albicans* and *C. glabrata*, with FICI values ranging between 0.375 and 0.5. These combinations notably reduced the antifungal MICs by 4‐ to 8‐fold. *S. calamintha* EO showed synergistic interaction against *C. albicans*, *C. glabrata*, and *C. krusei* (FICI = 0.5 and MIC gain = 4), and additive effect against *C. parapsilosis*, while *S. hortensis* showed synergistic interaction against *C. glabrata*, additivity against *C. albicans* and *C. krusei*, and indifference against *C. parapsilosis*. Regarding bacterial strains, the combinations of the investigated EOs and gentamicin generated more pronounced synergistic effects across all tested bacterial strains, with FICI values ranging between 0.252 and 0.5 (Table 5). Remarkably, all combinations tested displayed heightened synergistic effects against both the Gram‐negative *Escherichia coli* and *P. aeruginosa*, with an FICI of 0.25 and a significant reduction in antibiotic MICs by up to 512‐fold. These findings are in agreement with those reported by Vitanza et al. [20]., which showed that *S. montana* interacts synergistically with the antibiotic gentamicin against reference and clinical bacterial strains of *E. coli*, *S. aureus*, and *Listeria monocytogenes*. As far as we know, except for this study, the synergistic combinations of studied *Satureja* EOs with antimicrobial drugs have not been explored in previous research. Nevertheless, it is worth noting that the EOs under investigation are distinguished by the presence of key compounds known for their potent antimicrobial synergistic interactions with various antimicrobial agents. For instance, carvacrol, which is the predominant compound of studied *S. montana* and *S. hortensis* EOs, has been reported to possess high synergism with many conventional antibiotics, including amphotericin B and gentamicin [14, 15, 41].

**TABLE 4:** Fractional inhibitory concentration index (FICI) and MIC gain of amphotericin B (amph) combined with EOs obtained from studied *Satureja* species.

Essential oils	Strains	MIC amph (µg/mL)	MIC amph + EO	FIC (amph)	FIC (EO)	FICI	Gain
*S. alpina*	*C. albicans*	3.125	0.39	0.125	0.25	0.375	**8**
	*C. glabrata*	1.56	0.39	0.25	0.25	0.5	**4**
	*C. krusei*	0.78	0.39	0.5	0.25	0.75	**2**
	*C. parapsilosis*	1.56	0.78	0.5	0.25	0.75	**2**
*S. calamintha*	*C. albicans*	3.125	0.78	0.25	0.25	0.5	**4**
	*C. glabrata*	1.56	0.39	0.25	0.25	0.5	**4**
	*C. krusei*	0.78	0.195	0.25	0.25	0.5	**4**
	*C. parapsilosis*	1.56	0.78	0.5	0.25	0.75	**2**
*S. montana*	*C. albicans*	3.125	0.78	0.25	0.25	0.5	**4**
	*C. glabrata*	1.56	0.39	0.25	0.25	0.5	**4**
	*C. krusei*	0.78	0.78	1	0.25	1.25	**1**
	*C. parapsilosis*	1.56	1.56	1	0.25	1.25	**1**
*S. hortensis*	*C. albicans*	3.125	1.562	0.5	0.25	0.75	**2**
	*C. glabrata*	1.56	0.39	0.25	0.25	0.5	**4**
	*C. krusei*	0.78	0.39	0.5	0.25	0.75	**2**
	*C. parapsilosis*	1.56	1.56	1	0.25	1.25	**1**

**TABLE 5:** Fractional inhibitory concentration index (FICI) and MIC gain of gentamicin (gent) and EOs obtained from studied *Satureja* species.

Essential oils	Bacteria	MIC Gent (µg/mL)	MICgent + EO	FIC (gent)	FIC (EO)	FICI	Gain
*S. alpina*	*S. aureus*	0.312	0.002	0.007	0.25	0.257	**128**
	*M. luteus*	0.625	0.004	0.0625	0.25	0.3125	**16**
	*B. subtilis*	0.31	0.002	0.007	0.25	0.257	**128**
	*E. coli*	5	0.019	0.004	0.25	0.254	**256**
	*P. aeruginosa*	5	0.019	0.004	0.25	0.254	**256**
	*K. pneumoniae*	160	26.66	0.166	0.25	0.416	**16**
*S. calamintha*	*S. aureus*	0.312	0.078	0.25	0.25	0.5	**4**
	*M. luteus*	0.625	0.078	0.125	0.25	0.375	**8**
	*B. subtilis*	0.31	0.0006	0.002	0.25	0.252	**512**
	*E. coli*	5	0.019	0.004	0.25	0.254	**256**
	*P. aeruginosa*	5	0.019	0.004	0.25	0.254	**256**
	*K. pneumoniae*	160	5	0.031	0.25	0.281	**32**
*S. montana*	*S. aureus*	0.312	0.001	0.004	0.25	0.254	**256**
	*M. luteus*	0.625	0.156	0.25	0.25	0.5	**4**
	*B. subtilis*	0.31	0.004	0.015	0.25	0.265	**64**
	*E. coli*	5	0.019	0.004	0.25	0.254	**256**
	*P. aeruginosa*	5	0.009	0.002	0.25	0.252	**512**
	*K. pneumoniae*	160	20	0.125	0.25	0.375	**8**
*S. hortensis*	*S. aureus*	0.312	0.052	0.166	0.25	0.416	**16**
	*M. luteus*	0.625	0.078	0.125	0.25	0.375	**8**
	*B. subtilis*	0.31	0.001	0.004	0.25	0.254	**256**
	*E. coli*	5	0.009	0.002	0.25	0.252	**512**
	*P. aeruginosa*	5	0.019	0.004	0.25	0.254	**256**
	*K. pneumoniae*	160	10	0.0625	0.25	0.3125	**16**

## 3 Conclusions

The results of the conducted research indicate that there is diversity in both the chemical composition and antimicrobial activity among the investigated *Satureja* EOs. *S. montana* and *S. hortensis* EOs, which contain high levels of phenolic monoterpene carvacrol, displayed strong antimicrobial effects against all tested strains. Conversely, EOs from *S. calamintha* and *S. alpina*, rich in monoterpene ketone pulegone, exhibited relatively weaker antimicrobial activity. Interestingly, all *Satureja* EOs demonstrated significant synergy with the antibiotic gentamicin, especially against some common pathogenic bacteria, such as *Staphylococcus aureus*, *Bacillus subtilis*, and the two Gram‐negative *E. coli*, and *P. aeruginosa*. This finding suggests that EOs from these *Satureja* species could serve as potential adjuvants to these conventional antibiotics, offering novel strategies for enhancing their antimicrobial efficacy. These insights hold promise for the pharmaceutical industry, contributing to drug development and promoting the integration of *Satureja* EOs as complementary antimicrobial agents.

## 4 Experimental

### 4.1 Plant Materials and EO Analysis

Aerial parts of *S. alpina* and *S. calamintha* were harvested from their natural populations situated in the Oukaimeden region, while *S. montana* and *S. hortensis* were collected from an experimental parcel located in Oulad Dlim in the Marrakech Region (Table 1). All the plant materials were harvested during the full flowering period in the year 2022. The botanical identification of the plant material was confirmed by Pr. Mohamed Taleb Sghir, and voucher specimens (SATAL057, SATCA019, SATMO017, and SATHO012 assigned to *S. alpina*, *S. calamintha*, *S. montana*, and *S. hortensis*, respectively) have been deposited at the Laboratory of Microbial Biotechnologies, Agrosciences, and Environment, University Cadi Ayyad. EO extractions were performed in triplicate (3 × 300 g of dried plant materials) using hydrodistillation with a Clevenger‐type apparatus. The aerial parts of the plants were first dried in the shade at room temperature (≈ 25°C) until a constant weight was reached. The dried plant materials were then immersed in distilled water and subjected to hydrodistillation for at least 3 hours, ensuring complete recovery of the EO. The extracted EOs were separated from the aqueous phase, and dried over anhydrous sodium sulfate. The triplicates of each extracted EO were combined into a single sample and stored in amber glass bottles at 4°C until further analysis. The extraction yields were calculated as % (v/w) based on the dry weight of plant materials.

The qualitative and quantitative identification of the EO constituents was conducted using gas chromatography (GC) and GC coupled to mass spectrometry (GC‐MS) as previously described by Soulaimani et al. [42]. To identify the individual compounds of the chromatographic profile for each oil on a single analysis, the mass spectra were compared to authentic reference compounds where possible, and by reference to NBS75K and WILEY275 libraries, and a published terpene library [43]. The retention indices (RIs) were calculated relative to the retention times of a series of C_7_–C_30_ *n*‐alkanes, with linear interpolation, and compared with those of authentic compounds or published data.

### 4.2 Determination of the Antimicrobial Activity

#### 4.2.1 Microorganism Strains

Antimicrobial activity of the studied EOs was tested against four pathogenic clinically isolated Candida strains: *C. albicans* (CCMM L4), *C. glabrata* (CCMM L7), *C. krusei* (CCMM L10), and *C. parapsilosis* (CCMM L18), and six pathogenic bacteria: *S. aureus* (CCMMB3), *Micrococcus luteus* (ATCC 10,240), *B. subtilis* (ATCC 9524), *E. coli* (ATCC 8739), *Klebsiella pneumoniae* (Clinical isolate), and *P. aeruginosa* (DSM 50090).

#### 4.2.2 Antimicrobial Screening

The antimicrobial activity of *Satureja* EOs was evaluated using the agar disc diffusion and microwell dilution methods as described in the Clinical and Laboratory Standards Institute guidelines [44, 45]. For the agar diffusion test, sterile 6 mm diameter discs containing 10 µL of the EOs were applied to the surface of Sabouraud dextrose (SDA) or Mueller Hinton Agar (MHA) agar plates previously seeded by 0.1 mL of yeast or bacterial suspensions at 10^5^ and 10^8^ UFC/mL, respectively. All plates were kept at 4°C for 4 h to allow the EO diffusion before their incubation at 37°C for 24 h for bacteria, and at 28°C for 48 h for yeasts. Antimicrobial activities were evaluated by measuring the diameter of the IZs around the discs. All tests were repeated three times. Gentamicin (15 µg/disc) and amphotericin B (5 µg/disk) were used as positive controls. All assays were performed in triplicate. Regarding the microwell dilution method, 2‐fold serial dilutions of the EOs were prepared in 4% dimethyl sulfoxide and 100 µL of each dilution were added to microwells previously inoculated with 100 µL of yeasts or bacterial cell suspensions of 1–2 × 10^3^ and 10^8^ CFU/mL, respectively. The microplates were incubated for 18–24 h at 28°C for Candida strains and at 37°C for bacteria. The MIC was defined as the lowest EO concentration that inhibits the growth of the tested strains. To determine the MMC, 0.1 mL of clear wells that did not show growth during MIC assays were sub‐cultured on MHA or SDA depending on the strain type, and incubated in the same conditions described above. The MMC was defined as the lowest bactericidal and candidacidal EO concentrations. Gentamicin and amphotericin B were used as standard antibacterial and antifungal drugs, respectively.

#### 4.2.3 Synergistic Effect of *Satureja* EOs With Antimicrobial Drugs

Synergistic effects of *Satureja* EOs with antimicrobial drugs (gentamicin and amphotericin B), were carried out using the checkerboard method [46]. Briefly, 50 µL of each antimicrobial dilution was mixed in a microwell with 50 µL of EO dilution, then inoculated by 100 µL of cell suspensions of 10^8^ CFU/mL for bacteria and 1–2 × 10^3^ CFU/mL for candida. The microplates were incubated in the appropriate conditions described above, and the results were expressed in terms of an FICI using the following formula:

FICI=FICofEO+FICofantibiotic

with FIC of EO = MIC of EO in Combination/MIC of EO Alone

and

FIC of Antibiotic = MIC of Antibiotic in Combination/MIC of Antibiotic Alone

The FICI results were interpreted as: Synergism when FICI ≤ 0.5, additivity when 0.5< FICI ≤ 1, indifference when 1< FICI ≤ 2, or antagonism when FICI ≥ 2 [38].

The MIC gain of the antimicrobial drugs was calculated according to the following formula:

$$\begin{array}{ccc} \mathrm{MIC}\;\mathrm{gain}\; & = & \;\mathrm{MIC}\;\mathrm{of}\;\mathrm{antimicrobial}\;\mathrm{alone}/\\ & & \mathrm{MIC}\;\mathrm{of}\;\mathrm{antimicrobial}\;\mathrm{in}\;\mathrm{combination} \end{array}$$

## Author Contributions

**Imane Abbad**: writing–original draft preparation. **Bouchra Soulaimani**: writing–original draft preparation. **Imane Abbad**: methodology. **Bouchra Soulaimani**: methodology. **Mustapha Barakate**: methodology. **Marcello Iriti**: writing–review and editing. **Mustapha Barakate**: writing–review and editing. **Marcello Iriti**: conceptualization. **Mustapha Barakate**: conceptualization. All authors have read and agreed to the published version of the manuscript.

## Conflicts of Interest

The authors declare no conflicts of interest.

## Data Availability

The authors have nothing to report.
